# Reduction of inappropriate medication in older populations by electronic decision support (the PRIMA-eDS study): a qualitative study of practical implementation in primary care

**DOI:** 10.1186/s12875-018-0789-3

**Published:** 2018-07-09

**Authors:** Anja Rieckert, Christina Sommerauer, Anja Krumeich, Andreas Sönnichsen

**Affiliations:** 1 Institute of General Practice and Family Medicine, Faculty of Health, Witten/Herdecke University, Alfred-Herrhausen-Str. 50, 58448 Witten, Germany; 2 Department of Health, Ethics, and Society, Faculty of Health, Medicine, and Lifesciences, Maastricht University, Debyeplein 1, 6229 HA Maastricht, The Netherlands; 3 Division of Population Health, Health Services Research and Primary Care, School of Health Sciences, University of Manchester, Oxford Rd 176, Manchester, M13 9PL UK

**Keywords:** General practitioner, Evidence-based medicine, Computerized clinical decision support system, Deprescribing, Perceptions, Aged

## Abstract

**Background:**

Within the EU-funded project PRIMA-eDS (**P**olypharmacy in chronic diseases: **R**eduction of **I**nappropriate **M**edication and **A**dverse drug events in older populations by **e**lectronic **D**ecision **S**upport) an electronic decision support tool (the “PRIMA-eDS-tool”) was developed for general practitioners (GPs) to reduce inappropriate medication in their older polypharmacy patients. After entering patient data relevant to prescribing in an electronic case report form the physician received a comprehensive medication review (CMR) on his/her screen displaying recommendations regarding missing indications, necessary laboratory tests, evidence-base of current medication, dose adjustments for renal malfunction, potentially harmful drug-drug interactions, contra-indications, and possible adverse drug events. We set out to explore the usage of the PRIMA-eDS tool and the adoption of the recommendations provided by the CMR to optimise the tool and prepare it for its future implementation.

**Methods:**

In a qualitative study carried out in North Rhine-Westphalia, Germany, 21 GPs using the PRIMA-eDS tool within the PRIMA-eDS study were interviewed. Interviews encompassed the GPs’ attitudes regarding use of the electronic case report form and the CMR, their response to the recommendations, and the implementation of the tool into daily practice routine. The collected data were analysed applying thematic qualitative text analysis.

**Results:**

GPs found the patient data entry into the electronic case report form to be inconvenient and time-consuming. The CMR was conducted often outside practice hours and without the patient present. GPs found that the PRIMA-eDS CMR provided relevant information for and had several positive effects on the caring process. However, they encountered several barriers when wanting to change medication.

**Conclusions:**

It is unlikely that the PRIMA-eDS CMR will be used in the future as it is now as patient data entry is too time-consuming. Several barriers towards deprescribing medications were found which are common in deprescribing studies. Given the positive attitude towards the CMR, a new way of entering patient data into the PRIMA-eDS tool to create the CMR needs to be developed.

**Electronic supplementary material:**

The online version of this article (10.1186/s12875-018-0789-3) contains supplementary material, which is available to authorized users.

## Background

The geriatric population is growing in Europe [1]. Providing excellent care for older people with multimorbidity (i.e. multiple coexisting diseases in the same individual [2]) is complex and poses a challenge to general practitioners (GPs) in their daily routine [3, 4]. This challenge results from the many care components that need to be considered in a limited consultation time [4] in addition to the fact that easily accessible information on appropriate treatment in multimorbidity is lacking [3]. In order to treat multiple diseases physicians frequently prescribe several drugs. Taking a number of medications is known as polypharmacy [5], though exact definitions vary across the literature [6]. Polypharmacy can be problematic as it increases the risk of medication errors and may lead to adverse effects such as drug-related hospitalisations [7, 8].

Several strategies to optimise medication have been developed and tested in various settings over the past years, which can be divided into explicit or criterion-based measures (such as the Beers criteria [9]), implicit or judgment-based measures (such as the medication appropriate index [10]), and tools combining both approaches (such as the Australian Prescribing Indicators [11]) [12]. Such interventions have demonstrated improvements in appropriate polypharmacy and reductions in inappropriate prescribing, however, evidence for significant improvements of clinically relevant endpoints such as mortality or hospitalisations remains unclear [13–16]. There is a need to develop further strategies and to test them in large randomised controlled trials that have sufficient power to detect clinically relevant outcomes, and besides, asses the feasibility of the use of the strategies [16].

Within the health care system, an ideal point to optimise a patient’s medication is primary care [17]. However, many GPs feel uncertain about dealing with their multimorbid, older patients and desire more precise information about the benefits and risks of drugs and about patient relevant outcomes [3, 4, 18, 19]. A further difficulty is to deal with disease-specific guidelines as GPs feel uncertain about applying guidelines which focus on single diseases and often do not take the older age or the complexity of multimorbidity into account [3]. Besides, routines for assessing medication plans regularly do not exist [17]. Studies show that physicians would appreciate support to manage complex information [18], such as provided by computerised decision support systems (CDSS) [20].

CDSS have the capacity to help clinicians process complex clinical information [21]. Over the years, many different CDSS have been developed with some of them showing improved outcomes such as a reduction of serious medication errors and an increase in adhering to guidelines [21]. However, there have also been problems with CDSS such as the overriding of highly important alerts due to alert fatigue [21]. Besides, the mere provision of a CDSS does not guarantee its uptake. A systematic review on factors impacting on CDSS for prescribing concluded that there is no universal approach to successful CDSS implementation. Factors influencing the use and adoption relate to organisational factors such as the infrastructure and implementation, provider-related factors such as knowledge, training, current practice and preferences, patient-related factors such as patient and doctor-patient characteristics, and issues specific to the CDSS such as integration into the workflow [22].

Within the frame of the EU-funded PRIMA-eDS (**P**olypharmacy in chronic diseases: **R**eduction of **I**nappropriate **M**edication and **A**dverse drug events in older populations by **e**lectronic **D**ecision **S**upport) project, a CDSS was developed for GPs. The PRIMA-eDS tool aims to reduce inappropriate and non-evidence-based medication in the context of polypharmacy in older and chronically ill people by tailoring prescriptions of medications to the individual patient and thus maximising the patients’ health gain [23]. The recommendations of the tool are based on the highest standard of research evidence that is currently available [24]. The novelty of the PRIMA-eDS tool is that it provides a comprehensive medication review (CMR) based on current best evidence [25]. Basically, the PRIMA-eDS tool consists of two components. First, GPs or medical assistants type relevant patient data in an electronic case-report form (eCRF), which can be accessed online. Data collection includes: age, sex, height, weight, blood pressure, all drugs with a systemic effect (prescribed and over the counter), all diagnoses, current symptoms, several laboratory measurements, and frailty according to the clinical frailty scale [26]. Data should be up to date and be collected within a routine appointment. Once data entry is completed, the button “start medication review” needs to be clicked. The patient data are then sent via the internet to the EBMeDS service where they are evaluated and the CMR is created. Within a few seconds the CMR report (the second component of the PRIMA-eDS tool) opens in a new window. The GP can then review the CMR and bring the results together with his/her clinical expertise. There is also the option to print the CMR or save it as a pdf. The CMR consists of recommendations tailored to the individual patient to support the GP in clinical decisions and should not replace careful clinical consideration. The CMR is comprehensive and includes

- An indication check for all prescribed medications according to the diagnoses provided in the eCRF.
- A summary of laboratory test results that are relevant for the appropriate and safe use of the drugs on the medication list, and alerts if a result is outside recommended limits or if a repeat measurement may be due.
- Suggested evidence-based recommendations related to the medication. These can include, but are not limited to recommendations on: discontinuing drugs where there is no evidence for a positive risk/benefit balance, or where evidence points out that risks may outweigh benefits in older people; discontinuing drugs that are on lists of potentially inappropriate drugs for older people (PIM lists), such as the EU(7)-PIM list [27]; discontinuing drugs that have been used for a longer period than recommended in guidelines; discontinuing drugs that are not indicated on the basis of a small or questionable anticipated effect; ordering follow-up laboratory tests for monitoring and improving patient safety.
- Alerts related to drug prescribing in cases of renal malfunction.
- Alerts about known potentially harmful interactions between the patient’s drugs.
- Alerts about contraindications in case the patient has a recorded diagnosis that is a contraindication for one of his/her current drugs.
- Dose warnings in case daily doses exceed the recommended maximal levels according to the indication provided.
- A table listing the risks of nine standard adverse effects (anticholinergic effect, risk of bleeding, constipation, orthostatic dysregulation, QT-prolongation, renal toxicity, sedation, risk of seizures, serotonergic effect) possibly associated with each current drug.

**Fig. 1:**
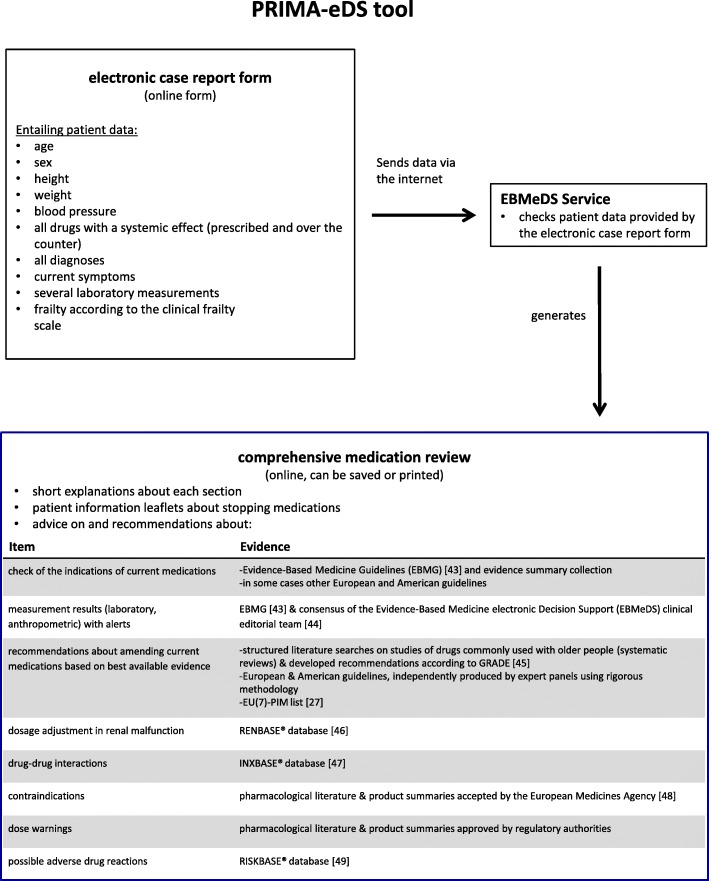
Components of the PRIMA-eDS tool [43–49]

**Fig. 2:**
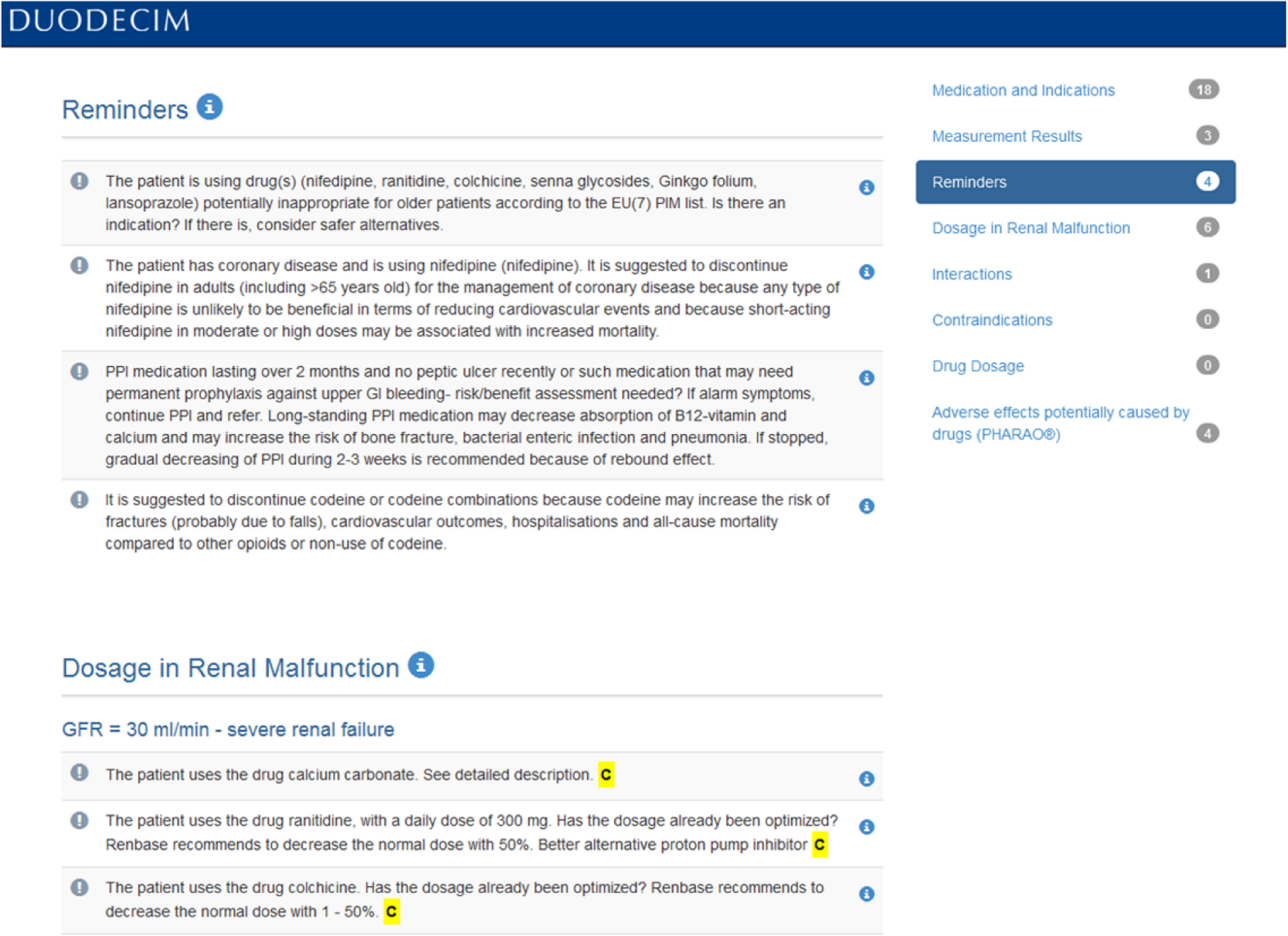
Screenshot from the Comprehensive Medication Review tool by Duodecim Medical Publications Ltd. showing recommendations about amending current medications and recommendations regarding dosing in renal malfunction

**Fig. 3:**
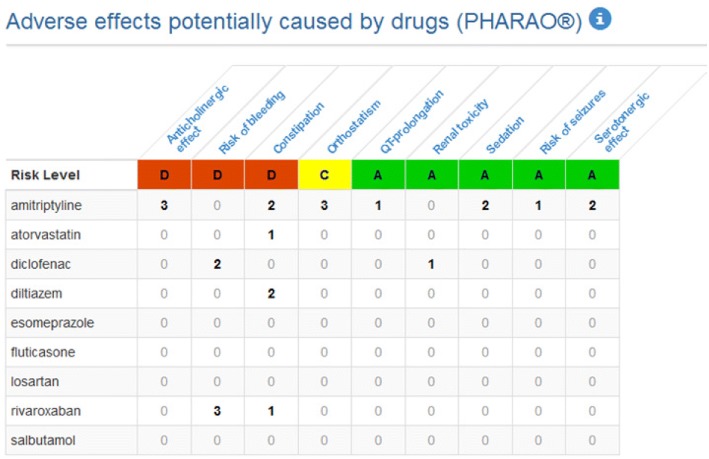
Screenshot from the Comprehensive Medication Review tool by Duodecim Medical Publications Ltd. showing the RISKBASE® table (former PHARAO®)

In this study we refer to all parts of the CMR as recommendations. It is at the discretion of the GP and the patient to decide whether to reduce, change or withdraw a medication or not. We recommended the GPs to make this decision in a shared decision-making process with the patient. Figure 1 depicts the two components (the eCRF and the CMR) of the PRIMA-eDS tool, and Figs. 2 and 3 depict excerpts of the CMR.

**Fig. 4:**
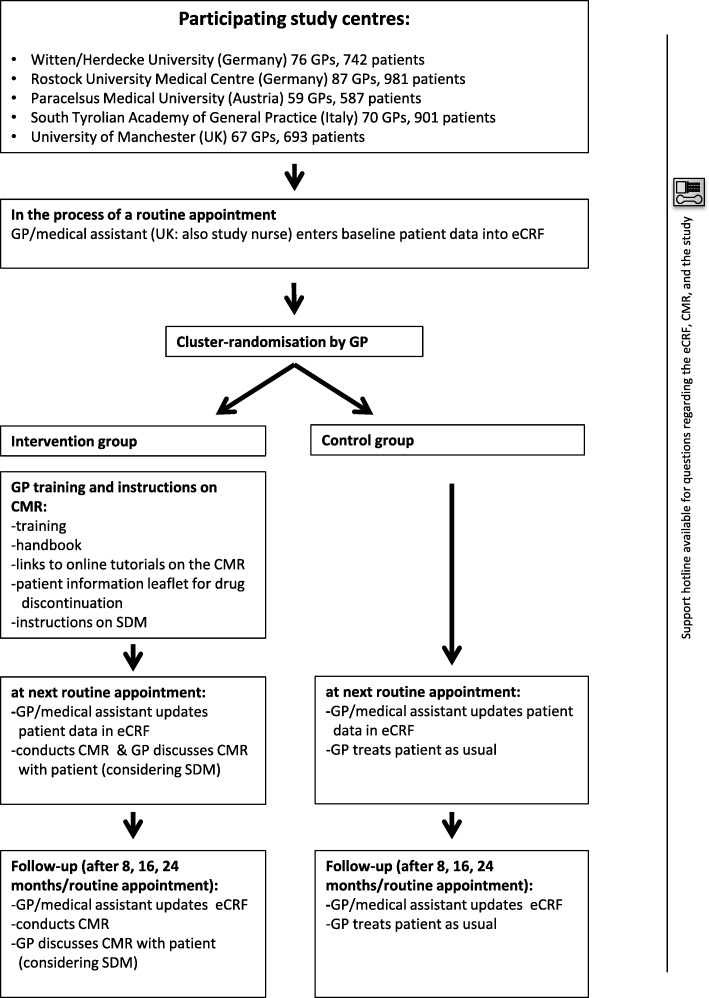
Course of the PRIMA-eDS study

The PRIMA-eDS tool is currently being tested in a pragmatic multicentre cluster randomised controlled trial in patients ≥75 years taking at least eight drugs. The primary outcome is a composite endpoint of first non-elective hospital admission or death during a two-year observation period. Three thousand nine hundred four patients and 359 GPs were included in the trial. Figure 4 outlines the course of the PRIMA-eDS study. Prior to the start of the randomised controlled trial, the PRIMA-eDS tool was piloted. More details of the PRIMA-eDS trial can be found elsewhere [25].

It is important to understand usage and acceptance of the PRIMA-eDS tool in order to optimise it so that it can be implemented into routine primary care practice, provided that it is effective and cost-effective. This exploratory study examines how GPs experienced the use of the PRIMA-eDS tool, how GPs adopted the recommendations provided by the CMR, and explores GPs’ ideas on the future implementation of the tool.

## Methods

An exploratory study design based on semi-structured interviews was adopted to gather an in-depth understanding of GPs’ experiences with the PRIMA-eDS tool. We report this study according to the Consolidated Criteria for Reporting Qualitative Research (COREQ) guidelines [28].

### Recruitment and data collection

We conducted in-depth interviews with GPs belonging to the intervention group of the PRIMA-eDS study centre of Witten/Herdecke University, Germany. GPs from the study centre Witten/Herdecke University come from cities and rural areas of the Ruhr valley in North Rhine-Westphalia, Germany. In total, the PRIMA-eDS study centre Witten/Herdecke University comprises 76 GP practices and 35 of them belong to the intervention group. GPs had been participating in the PRIMA-eDS study for up to 1 year when being invited. Using a random order list, we invited all GPs that had used the PRIMA-eDS tool at least twice. Semi-structured interviews with open-ended questions were undertaken to explore the use of the PRIMA-eDS tool and gather ideas on how the tool could be implemented into daily practice routine. We followed the concept of information power [29] and carried out interviews until we had sufficient information power fitting to the aim and analysis of our study as well as to our sample specificity and the generated quality of dialogue. Three interviewers with different backgrounds were trained and carried out the interviews: a research assistant of the PRIMA-eDS project with a master in public health (AR), a medical doctor working for PRIMA-eDS (CS), and a medical student with a bachelor in health communication who was not involved otherwise in the PRIMA-eDS study. All interviewers were not familiar with the participants. At the start of each interview, it was made clear that the interviewers were independent and that opinions in all directions could be expressed without any consequences. The interviews were carried out in the GPs’ own offices as this provided the GPs’ familiar surroundings where we expected the GPs to feel comfortable and open to talk about their experiences on a personal level. Furthermore, in this way we avoided possible travel-related burden for the interviewees.

Interviews were conducted in German. The interview questions covered the following topics: polypharmacy in everyday practice, using the eCRF, general overview of the comprehensive medication review, output of the CMR and how GPs responded to the recommendations, and the implementation of the tool into daily practice routine (see Table 1). Twenty-one GPs were interviewed between August 2015 and January 2016. Time of interviews ranged from 21 to 53 min. The characteristics of the interviewed GPs are presented in Table 2.

**Table 1:** Interview guide main questions

1. **Polypharmacy in everyday practice** *(Icebreaker)*	
Which role do polypharmacy patients play in your daily practice?	
2. **Using the eCRF**	
You have entered [number of] patients, what experiences did you have with the eCRF?	
3. **General overview of the CMR**	
What are your experiences with the PRIMA-eDS CMR?	
4. **Output of the CMR and how GPs responded to the recommendations**	
*Going through the CMR of one of the GP’s patient*	
What do you think about the various sections?	
What happens between reading the recommendations and the moment of discontinuing medication?	
How did the CMR influence you in treating your patients?	
5. **Implementation of the PRIMA-eDS tool into daily practice routine**	
Which benefits/barriers do you see in using the PRIMA-eDS tool in the future?	
Do you have any recommendations for further development?

**Table 2:** Characteristics of GPs (*N* = 21)

Characteristic	N (%)	Median (range)
Female	7 (33.3)	
Age (in years)		53 (41–65)
Years in practice*		16 (7–32)
Working in a single-handed practice	5 (23.8)	
Number of patients included in the main trial		11 (4–18)

*N = 19

### Data analysis

Interviews were recorded, anonymised and transcribed verbatim. For the analysis, all interviews were taken into account at full length. Thematic qualitative text analysis with an deductive-inductive approach was conducted to analyse the material as described by Kuckartz [30] consisting of seven steps. First, AR and CS independently read all transcripts and initially worked with the text by highlighting important passages and composing memos. Second, we developed the main topical categories from the interview guide, and third, conducted the first coding process by coding the entire material using the main categories. Fourth, we compiled all passages assigned to each of the main categories, and fifth, determined sub-categories inductively from the material. The sixth step was the second coding process: coding all of the data using the elaborate system. Thereafter, as a seventh step, we conducted a category-based analysis along the main topics. Two coders (AR and CS) independently coded the material. Any differences were solved by discussion. Contradictory data was taken into account. The software MAXQDA 12 was used in the analysis and coding process. Interviews were analysed in German. The coding scheme is presented in Additional file 1.

### Ethics

The study has been approved by the ethics committee of Witten/Herdecke University in July 2015 [no: 92/2015]. Written informed consent was obtained from the participants before the start of each interview. The anonymity and confidentiality of the participants was ensured by coding the participants as numbers (1–21) and by removing all identifiers during the transcribing process and for the presentation of the results. As a possible burden could arise for the GPs participating in the interviews in the form of an increased work and time expenditure, we compensated the time spent by an allowance for expenses. The GPs were able to terminate the interviews at any time, though this did not happen.

## Results

We present our results according to the adoption of the intervention: the eCRF, the CMR, the adoption of the recommendations, and future implementation. Selected quotations are presented to illustrate answers from various respondents.

### Adoption of the intervention: The eCRF

The most important experience with entering patient data (see Fig. 1 for a description of the variables) into the eCRF was that it was considered to be time-consuming. After a period of familiarisation and when updating data, utilisation became considerably easier and faster:

*In the beginning it took a while, but then it went pretty quickly. […] For the first one I took 45 minutes I think and in the end it took me ten minutes. (GP 14).*

Initially, operating the eCRF was perceived as unusual.

*In the beginning you had to familiarise yourself a bit with this programme first. This required some getting used to it, and for sure the error rate was high at first, too. But somehow in the course of time you learned how to use it. (GP 21).*

GPs mainly filled out the eCRF themselves as not all variables were routinely documented in the practices. Some GPs delegated data entry to healthcare assistants. Not all GP practices had internet access, which complicated data entry. In these cases, data entry was done at home. Data entry was not always carried out while the patient was present as for example this GP outlines:

*I added his medication later, without him being there. If you sit there and type something, it can be rather impersonal. I don’t like doing that too much. (GP 14).*

Switching between the eCRF and medical practice software was perceived as being inconvenient. Sometimes content from the practice software needed to be printed out or written down in order to add it to the eCRF. Not always was the patient asked about symptoms and medical conditions but they were rather transferred from the patients’ files or estimated.

*I put in the important facts to the best of my knowledge, but I often thought that I didn’t talk about it. (GP 8).*

Furthermore, GPs complained that sometimes technical problems occurred:

*Once or twice I wanted to go back [to the patient in the eCRF], but it said that I can’t. (GP 5).*

### Adoption of the intervention: The CMR

#### Usage

The medication review was utilized in different situations, depending on technical possibilities and time opportunities. In the GP practices, medication reviews were carried out with and without the patient:

*Sometimes I did it after they left, the next day or the same day or during the break or something like that. Sometimes I did it with the patients, provided they were mentally fit. (GP 16).*

Some GPs conducted the CMR without the patient and even outside practice hours. Some printed the CMR. Some thought about the recommendations and made notes about possible changes in the patient chart:

*I did it after the patient was here. […] I usually make notes and I put them on my desk. Until I discuss them with my patients […] that can take a few days. (GP 17).*

*On the weekends here. It’s impossible to do it in between the practice hours. […] I printed out the medication plan and noted down what [medication changes] I wanted to discuss. (GP 8).*

*So, I usually don’t use it in the presence of the patient, but when the patient is present in the practice. When the patient is waiting in the treatment room. Then I call up [the CMR] and then I go to [the patient]. (GP 13).*

Few GPs performed the medication review at home, made notes and discussed adjustments at a convenient opportunity with the patient. Reasons for carrying out the CMR at home were not having internet access at the practice and not finding time during the day in the practice:

*At home […] because I’m not online here. So I looked through it and then I noted for each patient down the recommendation from your list of suggestions. And then I thought about which of them I should implement and what things I keep in spite of the recommendation from the list. (GP 12).*

Sometimes patients were called into the practice to make changes in the medication. Rarely, they were informed about the results via phone. Usually changes were discussed during the next consultation:

*[I] wrote a memo “change study this to that”. [… I] then put it on my desk, then it takes two, three weeks until eventually I find a small hole and then I changed it. (GP 2).*

#### Experiences with the patient

Most GPs interviewed in our study talked about and discussed medication changes as a result of the CMR with their patients:

*I discuss it, I propose it [to the patient] and explain that this and that is the reason and that I read this [in the CMR of the] study and explain what could happen, the reason, [and ask] should we give it a try? (GP 2).*

However, not all discussed the recommendations with their patients as this GP outlines:

*Discussing is a bit difficult, it depends, and the thing is that those [patients that are in the study] are mostly not the kind of people that value discussions. […] They don’t like it, they’re not used to discuss extensively why one should […] change something. […] Older people, sick people, they normally accept what one offers them. It rarely happens that they ask ‘why’. (GP 9).*

GPs felt that the majority of patients taking part in the trial reacted in an open-minded and cooperative way towards recommended changes in medication. Some patients were happy about having to take fewer drugs.

*They are happy. Those people who didn’t want something like this; they are simply not part of the study. So, when I tell [the patient] that this medication check recommends this and that, then they are actually quite positive. (GP 7).*

Patients sometimes were scared of negative effects when changing long-term medication and feared that changes could rather be due to cost-cutting measures than to the objective of improving care.

*Generally, these are older patients and to convince them that certain drugs, that we need to change them, that’s not always easy. […] Patients are anyway quite worried because of these discount contracts [between the pharmaceutical companies and] the health insurances. (GP 18).*

GPs described the patients taking part in the trial as already being closely connected with them, and they had the impression that some patients liked to get more attention due to the extensive care.

*The patients, they were really enthusiastic, they enjoyed getting a lot of attention and felt properly checked up, figuratively spoken, concerning their drugs. Those [patients] really left beaming with joy. (GP 15).*

#### Expenditure of time

The estimated time needed to read the CMR varied (5–20 min). The question whether the CMR was too extensive for the consultation hours led to mixed responses:

*It [the time required] is relatively little. But on the other hand also still too much for me to do it directly during the daily business. […] If I had to do it by hand, I’d take forever to finish. (GP 17).*

*That was not time-consuming at all. (GP 11).*

Especially retrieving additional information provided by the tool was perceived as being too time-consuming. Some GPs mentioned that they hoped for a learning effect that would shorten expenditure of time in the future.

*In the longer term it is a tool which might tell you for the next patient, man, that’s not a very good combination, so it creates a learning effect as well. Therefore the time spent in relation to the benefit will clearly get better at some point. (GP 1).*

#### Evaluation of the CMR

The recommendations provided by the CMR served to update and complement existing knowledge.

*Prior knowledge pre-existed in many ways, but especially for example with interactions of psychotropic drugs, of neurological drugs, risk of haemorrhage in SSRIs and these things, these are not always that [present]. Risk of falling, especially things which are relevant to older people, that is helpful information for sure. (GP 1).*

GPs were positive about the CMR. The CMR was described as being useful, containing trustworthy and well-chosen recommendations that support the GP:

*You can’t possibly have all that in mind and it can be very helpful to be shown everything that you should basically take into consideration. (GP 17).*

Points of criticism for instance were that recommendations were hard to realise and that the medication review was ‘too tame’ as this GP reports:

*So I think it’s good to actually think about critical stuff and also to be made aware of it, but […] it is very ‘pacific’ [in the sense of not aggressive enough regarding recommendations to discontinue drugs]. (GP 13).*

### Adoption of the recommendations

Most GPs reported having changed medication due to the recommendations:

*[I] did change quite a bit and also discontinued [some drugs] and it didn’t affect the treatment of the patients negatively. Rather positively, because I tried harder to notice the one or other. (GP 8).*

*I made changes for almost everyone; I think except for one, I think I’ve changed something based on the recommendation dose reduction with reduced renal functioning. (GP 4).*

Sometimes, GPs felt that their reductions were only minor, such as that they had mainly reduced doses and only occasionally discontinued drugs.

*Mainly small changes. To some extend only in between one group of drugs, for instance simvastatin instead of atorvastatin. One time it was about reducing the dosage by half because of some renal function. I would not rate it that high right now. (GP 12).*

Some GPs reported that they were not able to follow any of the recommendations:

*Despite the medication check, I could hardly discontinue anything. (GP 13).*

At times, discontinued medication had to be restarted. However, in these cases the GPs were glad to have tried the discontinuation because then the decision to prescribe the medication was made more consciously and the necessity of the medication was confirmed.

*Unfortunately that didn’t work. But here I have to say, this really is something where one has to accept it, if one says, there’s no other way. This is of course a deliberate thing. What is important is that one simply always does everything deliberately and that one can justify why one’s doing something. (GP 14).*

Several GPs reported well-tolerated and lasting changes in medication.

*We also followed that and it actually worked out well. People are also coping fairly well with less or with an adjustment. (GP 8).*

Incorporation of the recommended changes was carried out gradually and needed time:

*I wanted to somehow slowly reduce. It cannot be stopped all of a sudden. (GP 20).*

#### Barriers to adopt recommendations

GPs expressed a multitude of reasons that hindered them in following recommendations (see Table 3 for quotes).

**Table 3:** Barriers to following the recommendations from the GPs’ point of view

Reasons for not following the recommendations	Quotes
Alternatives and recommendations had already been tested and the GP and/or the patient felt that this was not the optimal way of treatment.	It's a long way making that decision and once it’s made and then it is an important drug. I don’t care if there is a contraindication, he’ll get it nevertheless. (GP 19)
	Why in this patient I won’t follow the recommendations is that it has already been tried out in the past. (GP 3)
The GP regarded the medication as being necessary.	Out of the multimorbidity of the people, it is inevitable that one gives them [the drug]. (GP 6)
The GP and/or the patient had other priorities compared to the PRIMA-eDS tool.	Then the patient decides for me. From a certain age on it is about the quality of life. (GP 10)
	Concerning diclofenac for the older patients it simply is like that, he just doesn’t want [to discontinue the drug] and says, “you can’t take this away from me. [I am] free of pain for the first time in 7 years. I need that.” (GP 10)
The GP feared that changing medication could get complex.	In case of a patient for whom this medication works so well, in inverted commas, over such a long period of time I won’t change anything. This would just rock the boat. (GP 3)
The GP had been prescribing the medication for years and lacked motivation to reconsider.	And that is simply a drug that the patient is using for 30 years now and under which she is well managed concerning her blood levels. [And] as mentioned leading a life with very little hardship with over 90 years. I would not touch it, that is [a case of] ‘never change a winning team’, therefore these are things I wouldn’t change. (GP 12)
The GP did not want to diverge too far from a standard of therapy (guidelines).	So you have to ultimately stick to the general guidelines, because if you go there now radically, then you contravened the guidelines of the professional societies. It’s difficult. (GP 9)
The GP found the recommendation to be new and not comprehensible.	I’ve never heard that before, it somehow was completely new to me and so I ignored it. (GP 16)
The GP considered the recommendation as not applicable to the individual patient.	Where I say that the patient is biologically younger. (GP 1)
The GP found that the patient was a barrier to discontinue medications.	The patient won’t cooperate. If there wasn’t the patient, everything would be easier. (GP 15)
The prescription was made by another medical specialist and the GP did not want/ did not dare to change it.	Who is responsible for which prescription. The things I do not prescribe, the four medications I do not prescribe, the four psychotropic drugs, I can’t change that. (GP 7)
It seems that due to the infrastructure medication changes resulting from the CMR could have been delayed or even forgotten.	This actually is a relatively long process, as I don’t have internet access here. […] I print it [the CMR] and make notes. […] Then I wait until the patient comes again. But I have [a study patient] who doesn’t come very often and then it's difficult. (GP 2)

##### Prescriber factors

One reason was that GPs prioritized differently (e.g. pain relievers) compared to the PRIMA-eDS CMR or regarded medication as necessary and alternatives had already been tested. Sometimes, GPs did not follow recommendations as they feared that changing medication could get complex. Frequently they had been prescribing the medication for years and lacked motivation to reconsider it or did not want to diverge too far from a standard of therapy (guidelines). Sometimes GPs found the new recommendations not comprehensible or considered the recommendations as not applicable to the individual patient who was perceived biologically younger (see Table 3 for quotes).

##### Patient factors

At times, also the patient was perceived as a barrier. GPs felt that the willingness to change medication depended on the actual medication and priorities of the patient (e.g. pain relievers vs. antihypertensives). GPs reported that sporadically, patients postponed implementing medication changes (e.g. to after a holiday). Furthermore, it was described that some patients faced a dilemma: the wish to take fewer drugs but little willingness to change lifestyle (see Table 3 for quotes).

##### External factors

GPs were reluctant to discontinue medication prescribed by other medical specialists without contacting them. Contacting the specialist to change medication, however, took additional effort and GPs feared that it would be difficult to reach a consensus as the specialists often have a different viewpoint (see Table 3 for quotes).

##### Infrastructural factors

It seems that a further barrier was that the CMR was sometimes conducted without the patient being present in the practice (e.g. outside of practice hours) and thus medication changes resulting from the CMR could have been delayed or even forgotten.

#### Other effects resulting from the CMR

Besides changes in medication, the CMR generated further effects (see Table 4 for quotes).

**Table 4:** Effects of the CMR apart from medication changes

Effects of the CMR	Quotes
The CMR stimulated GPs to critically reflect on the medication more than usual and to make more conscious decisions.	Of course you engage more intensively with the patient and what he actually has to swallow. (GP 1)
	With other things I looked them up again and assured myself. It really is a push to grapple with things again that one should actually be mastering. (GP 18)
The CMR increased GPs’ awareness of risks associated with drug use in polypharmacy patients.	What is good about it, of course, it makes me aware again and again. (GP 4)
The CMR supported GPs in the dialogue with other medical specialists as the CMR provided good evidence for the GPs’ decision.	I can now say that the European study has recommended it. (GP 10)
GPs were able to transfer CMR results to patients outside of the study.	[I] changed quite a bit and also discontinued [some drugs] and it didn’t affect the treatment of the patients negatively. Rather positively, because I tried harder to notice the one or other and also with patients not participating in the study. (GP 8)

##### Treatment

The recommendations stimulated the GPs to critically reflect on the medication more than usual and helped to make a more conscious decision. Due to the CMR, the GPs became aware of risks and got a better sense about them. Some recommendations were transferred to patients not participating in the study (see Table 4 for quotes).

##### Communication

The recommendations supported the GPs in the dialogue with other medical specialists and patients as the GPs became more confident and better at explaining the results (see Table 4 for quotes).

### Future implementation

Overall, the interviewed GPs would like to use the CMR in their everyday practice provided that the data entry will be omitted by interlinking the tool with the electronic health record (EHR) so that the CMR becomes easily accessible:

*It has to be included in every software and then it is easy and then it will also be used. (GP 19).*

Nevertheless, according to the GPs, several hindering as well as enhancing factors exist for the future implementation. Many of them have already been named in the sections adoption of the intervention: the eCRF and the CMR.

Possible barriers to implementing the CMR in the future were: time, the required internet access, and possible costs (without being reimbursed). GPs worried about possible technical problems, and about ensuring data security (see Table 5 for quotes).

**Table 5:** Possible barriers to the future implementation from the GPs’ perspective

Possible barriers	Quotes
GPs worry that a possible barrier for the future use of the PRIMA-eDS tool could be…	
the time required.	If I should benefit from this in any way, it can’t go beyond the limitations of a certain time frame. (GP 12)
the required internet access (for those that are not connected yet).	Well we don’t have any internet access. (GP 9)
possible costs without being reimbursed.	It depends somewhat on the price. (GP 17)
the technical implementation and data security.	Besides [technical problems] none. (GP 5)
	If data security is given, none. (GP 3)

By implementing the PRIMA-eDS tool in the GPs’ daily routine, GPs would expect an optimisation of medication, an improvement in patient safety, a quick way of checking medication, and direct feedback when prescribing medication. Furthermore, GPs conceived the tool’s independence from the pharmaceutical industry and being free of commercial advertisements as strengths (see Table 6 for quotes).

**Table 6:** Possible enhancing factors for the future implementation from the GPs’ point of view

Possible enhancing factors	Quotes
GPs consider a strength for the future implementation of the PRIMA-eDS CMR that…	
it supports them in optimising medications and improving patient safety.	Yes, in first place patient security. That one really protects the patient from being harmed by drugs. (GP 14)
	Certainly reduces mistakes. (GP 10)
it provides a quick way of checking medications.	That one really can quickly control a patients’ [prescriptions], especially when he’s taking multiple drugs or a rare combination, that one then really succeeds in checking during everyday practice. (GP 17)
they can get direct feedback when prescribing a medication for the first time.	I think it’s great. That one might not take the wrong drug when making a new prescription but that one already sees a warning. (GP 8)
it is free of commercial advertisement and independent from the pharmaceutical industry.	Yes, I could imagine [to use it], especially if it's free of commercial advertisements and not sponsored by the pharmaceutical industry. (GP 16)

## Discussion

Our findings suggest that the PRIMA-eDS tool will not be used in daily practice outside of the study as it is too time consuming, especially with regard to entering patient data. On the other hand, GPs really value the CMR to be useful as it summarises important information regarding evidence-based and appropriate drug-treatment and stimulates the GP to critically reflect prescriptions. The GPs interviewed in our study clearly state that they would like to use the CMR in daily practice if the time-consuming entry of patient data were omitted.

### Comparison with existing literature

GPs named a multitude of reasons for not following the recommendations. Barriers to deprescribing (i.e. the process of withdrawing medications [31]) have been widely discussed in the literature. Several studies described the reluctance of GPs to interfere with medication prescribed by a colleague [3, 17–19, 32–34]. Furthermore, Schuling et al. showed that stopping a medication is not always easy and depends on the patients’ preferences (especially pain medication). A further barrier is that GPs feel guilty if they do not adhere to guidelines [18]*.* Some GPs also fear a potential for litigation when deprescribing medication [19].

In the literature, further barriers to deprescribing medication or using CDSS exist, such as that some GPs feel uncomfortable with reading and communicating measures of risk [18, 35]. These barriers were not brought up in our study. The PRIMA-eDS tool does not use numbers (like absolute or relative risks and numbers needed to treat) in the main message of the recommendations, making an understanding of risks easier. Such numbers were only shown when a GP clicked on a button for “further information”.

We recommended to the GPs to discuss the CMR with their patients in a shared decision-making process and gave instructions on how to do so. Most GPs stated that they involved the patient in the decision-making process of possible medication changes, though it seems that the actual way of discussion varied. Besides, we do not know how satisfied the patients really were as previous studies have shown that even though GPs rated their communication as adequate or very good, patients were not satisfied [36]. While it seems that GPs often involved their patients, it was also raised that there were older patients that did not want to be involved in the decision-making process. Previous research has shown that the desire for a shared decision-making process differs among older people. There are those patients that want to be involved, however, there are also those that do not want to be involved and simply accept the GPs’ decision [37]. Here again, we do not know how satisfied the patients were.

A key issue which was prominent throughout the main categories formed by our analysis was time. Time constraints as a barrier to use CDSS have been widely discussed in the literature [22, 35, 38–40]. Other barriers for implementing CDSS in the literature were lack of basic IT skills [35], earlier experience of dysfunctional computer systems in health care, and general resistance towards changes in practice [20]. These factors were not mentioned in our interviews, which might have to do with the selection of GPs and that our study was conducted several years later than most of these other studies.

### Implications for future implementation and use

For the implementation of the PRIMA-eDS tool, the whole process of entering patient data needs to become more time efficient. Future developments should focus on linking the CMR to the existing EHR as generally integrated systems are more likely to succeed than “stand alone” systems [22, 41]. This of course requires EHR to be up to date, which often is not the case in daily routine. In Germany, linking EHR to the CMR will not be easy to realise due to the multitude of software providers. Certainly, the CMR will not be able to unfold its full potential in case patient data is not complete or not completely up to date. Still, the CMR can be beneficial even with incomplete data. For instance, if only some medications are entered, the medication check can still be performed for these drugs. On the other hand, incomplete data could also lead to false alerts and reminders or to a lack of necessary alerts. This in turn could compromise the trustworthiness of the system, as encountered by Koskela et al. [42]. Thus, efforts to enhance patient data entry seem indispensable.

The PRIMA-eDS tool can only be used in daily routine when the GP practice is connected with the internet. Even though internet access in GP practices is common in Germany, there are still some practices that do not have it. Due to the ongoing digitalisation in health care systems, we expect that internet access will be available in most practices in the near future. Besides, for the implementation it needs to be ensured that possible costs are remunerated and that data security remains ensured.

### Strengths and weaknesses of the study

The GPs participating in this study are a selection of GPs who voluntarily participated in the PRIMA-eDS study as well as in this qualitative investigation. We expect them to be more interested in electronic decision support, the topic of polypharmacy, and to be more engaged compared to their peers. Still, we were able to interview GPs of various ages and different experience levels which provided the possibility to examine a multitude of views and experiences. The interviewed GPs were living in North Rhine-Westphalia, Germany, and thus there are some limitations to the generalisability of findings in other settings. The interviews were conducted within the first year of the study, and thus, the GPs were not able to have long-term experience with the PRIMA-eDS tool.

A further limitation is that we already knew due to our pilot study that the data entry into the eCRF was considered to be too time-consuming; however, we did not find any other way to circumvent it in the frame of the PRIMA-eDS project.

Data collection and analysis of the results was conducted by the research team consisting of multiple professions. Two of the interviewers were part of the PRIMA-eDS project team, which could possibly have biased the results in favour of the PRIMA-eDS tool, but we also involved one interviewer who was not involved in the PRIMA-eDS project otherwise to minimise this possible bias. Coding was done by two coders in order to enhance credibility of the findings.

## Conclusion

Entering patient data in the eCRF is too time consuming, and thus, it is unlikely that the PRIMA-eDS tool will be adopted in daily practice as it is now. GPs were positive about the CMR as it summarises important information regarding drug-treatment and stimulates the GP to critically reflect on prescriptions. Medications were reduced to some extent. However, various barriers to deprescribing medications remain, such as that the GP and/or the patient prioritised differently, the GP regarded the medication as necessary, the GP feared that changing medication could get complex, the GP found the recommendation not applicable to the patient, and the GP did not want to interfere with medication prescribed by a colleague. Given the positive attitude towards the CMR, it seems like it is worth to develop a way for easier patient data entry or direct implementation in existing EHR, providing that the CMR proves to be effective and cost-effective.

## Additional file

Additional file 1: Coding scheme. (PDF 26 kb)

## Abbreviations

CDSS: Computerised decision support system
CMR: Comprehensive medication review
eCRF: Electronic case-report form
EHR: Electronic health record
GP: General practitioner
PRIMA-eDS: Polypharmacy in chronic diseases: Reduction of Inappropriate Medication and Adverse drug events in older populations by electronic Decision Support
