# Supplementary material for: Reduction of inappropriate medication in older populations by electronic decision support (the PRIMA-eDS study): a qualitative study of practical implementation in primary care
Source: BMC Fam Pract. 2018 Jul 9;19:110. doi: 10.1186/s12875-018-0789-3 (PMC6038343; doi:10.1186/s12875-018-0789-3)
Supplement: Supplementary file 1 — Coding scheme. (PDF 26 kb) [file 12875_2018_789_MOESM1_ESM.pdf]

**Main category**	**Sub category**
Adoption of the intervention: the eCRF	Usage
	Entering data into the eCRF
	Effort
	Technical problems
Adoption of the intervention: the CMR	Usage
	Experiences with the patient
	Expenditure of time
	Evaluation of the CMR
Adoption of the recommendations	Medication changes
	Barriers
	Other effects resulting from the CMR
Future implementation	Future utilization
	Enhancing factors
	Hindering factors
